# Prognostic impact of pathological complete remission after preoperative irradiation in patients with locally advanced head and neck squamous cell carcinoma: re-analysis of a phase 3 clinical study

**DOI:** 10.1186/s13014-019-1428-4

**Published:** 2019-12-12

**Authors:** Kai Wang, Junlin Yi, Xiaodong Huang, Yuan Qu, Jingwei Luo, Jianping Xiao, Shiping Zhang, Yuan Tang, Weixin Liu, Guozhen Xu, Li Gao, Zhengang Xu, Shaoyan Liu, Xiaolei Wang

**Affiliations:** 1 Department of Radiation Oncology, National Cancer Center/Cancer Hospital, Chinese Academy of Medical Sciences and Peking Union Medical College, Beijing, China; 2 Department of Radiation Oncology, Peking University Cancer Hospital and Institute, Beijing, China; 3 Department of Head and Neck Surgery, National Cancer Center/Cancer HospitalChinese Academy of Medical Sciences and Peking Union Medical College, Beijing, China

**Keywords:** Head and neck squamous cell carcinoma, Preoperative radiotherapy, Concurrent chemoradiotherapy, Pathological complete remission, Prognosis

## Abstract

**Purpose:**

The purpose of this study was to determine the associations between pathological complete remission (pCR) and clinical outcomes in patients with locally advanced head and neck squamous cell carcinoma (HNSCC) who received preoperative radiotherapy or chemoradiotherapy in a phase 3 clinical study.

**Methods:**

A total of 222 newly diagnostic stage III/IVM0 HNSCC patients were randomly assigned to a preoperative concurrent chemoradiotherapy group (*n* = 104) or preoperative radiotherapy alone group (*n* = 118). Over a mean follow-up of 59 months, 72 patients were defined as non-responders to preoperative therapy and subsequently underwent resection of the primary lesion with or without neck dissection. The relationship between the pathological tumor response of the primary lesion and treatment prognosis was analyzed. Kaplan–Meier and Cox regression multivariate analyses were performed to evaluate the impact of pCR on local control (LC), overall survival (OS), progression-free survival (PFS), and distant metastasis-free survival (DMFS).

**Results:**

Among the 72 non-responders, 25 patients, 10 in the chemotherapy group and 15 in the radiotherapy group, achieved pCR. The 5-year LC, OS, PFS, and DMFS of pCR patients and non-pCR patients were 93.2% vs. 67.7% (*p* = 0.007), 83.3% vs. 39.7% (*p* = 0.0006), 76.1% vs. 44.0% (*p* = 0.009), and 90.4% vs. 56.3% (*p* = 0.005), respectively. In multivariate analysis, pCR is also an independent prognostic factor in prognosis, with statistically significant differences.

**Conclusion:**

pCR after preoperative radiotherapy or concurrent chemoradiotherapy is a good prognostic factor in locally advanced HNSCC.

**Trial registration:**

Number:ChiCTR-TRC-114004322

Date:05 Mar, 2014

## Introduction

Although surgery followed by radiotherapy and/or concurrent chemoradio- therapy has been the major treatment choice recommended by the NCCN guideline for treating locally advanced head and neck squamous cell carcinoma (HNSCC) for many years [1], the outcome with this regimen is still limited by a low 5-year survival rate, which remains < 40% [2, 3]. In recent years, several researches have suggested that preoperative radiotherapy and preoperative concurrent chemoradiotherapy may improve the overall survival (OS) compared to surgery alone in patients with head and neck cancer [4–6]. The advantages of preoperative radiotherapy or preoperative concurrent chemoradiotherapy for head and neck cancer include down-staging, increased resectability rate, etc. Further evidence from several phase 2 studies have shown that preoperative concurrent chemoradiotherapy provides excellent treatment outcomes by leading to a pathological complete response rate ranging from 35 to 61% and an increased 5-year OS rate of up to 70–81.5% [5–8].

Several authors have suggested that the pathological response to preoperative radiotherapy or chemoradiotherapy is a valuable prognostic factor for local control and OS [9, 10]. In one of our randomized phase 3 studies, we evaluated the role of adding concurrent cisplatin to preoperative radiotherapy in the treatment of locally advanced HNSCC [11]. In this study, 222 eligible patients were randomly assigned to a preoperative radiotherapy group (*n* = 118) or a preoperative concurrent chemoradiotherapy group (*n* = 104). The results revealed that preoperative concurrent chemoradiotherapy led to a significantly improved distant metastasis-free survival (DMFS) compared to preoperative radiotherapy alone. According to the protocol, non-responders (defined by < 80% reduction of the primary lesion after preoperative treatment), subsequently underwent resection of the primary lesion with or without neck dissection depending on the nodal status. In total, 72 non-responders (33.8%) were observed in this phase 3 study.

In the present study, we analyzed the relationships between pathological complete remission (pCR) and clinical outcomes in these 72 non-responder patients.

## Materials and methods

### Patients

The details of the study design and data collection have been published in our previous study [11]. In brief, from September 2002 to May 2012, a total of 222 HNSCC patients were enrolled in this phase 3 study, among which 104 patients were assigned to the preoperative concurrent chemoradiotherapy group and 118 patients were assigned to the preoperative radiotherapy group.

### Treatment

All these patients received radiotherapy with either two-dimensional or intensity-modulated radiotherapy (IMRT). Patients assigned to the chemoradiotherapy group additionally received concurrent chemotherapy with 30 mg/m^2^ cisplatin weekly. The tumor response was assessed by computed tomography (CT) and/or magnetic resonance imaging (MRI) and endoscopy examination at the end of the 5th week (50 Gy). Non-responders (defined as < 80% reduction of the primary lesion volume) underwent resection of the primary tumor and modified neck dissection within 4–6 weeks after the completion of preoperative treatment. Seventy-two patients were non-responders, including 35 patients in the preoperative radiotherapy group and 37 patients in the chemoradiotherapy group.

### Pathological response analysis

The surgical samples of all clinical non-responders were assessed pathologically, and patients were classified according to the pathological response as the pCR group and non-pCR group, depending on whether residual tumor cells remained in the surgical sample or not.

### Follow-up

After completion of the treatment plan, patients were followed up for a minimum of 5 years or until death, with a regular frequency: at 1 month, every 3 months for the first 2 years, every 6 months for the 2–5 years after treatment, and every year thereafter.

### Statistical analysis

In our previous study, we revealed that in a subset of patients with primary tumors of the larynx-hypopharynx, preoperative chemoradiotherapy significantly improved the PFS and DMFS, and also provided a borderline benefit in OS in compared with preoperative radiotherapy. Therefore, in the current study, a sub-analysis comparing clinical outcomes in patients with larynx-hypopharynx primary tumors was performed.

Log-rank test was applied to compare the differences in baseline characteristics between pCR and non-pCR patients. The LC, OS, PFS and DMFS were calculated by the Kaplan–Meier method. The multivariate analysis were by Cox regressive analysis. All tests were two-sided and *p* < 0.05 was considered statistically significant. All analyses were conducted using SPSS 21.0 software (IBM Corporation, Armonk, New York, U.S).

## Results

### Baseline characteristic between different pathological responses

The total of 72 non-responders included 37 patients in the preoperative concurrent chemoradiotherapy group (*n* = 104) and 35 patients in the preoperative radiotherapy group (*n* = 118). Among these 72 non-responders, 25 patients (34.7%) achieved pCR, including 10 patients (10/37) in the chemoradiotherapy group and 15 patients (15/25) in the radiotherapy group (Table 1). There were no significant differences between the pCR and non-pCR groups in terms of primary tumor site, tumor staging (T, N stage), grades of clinical group, and treatment technique (all *p* > 0.05).

**Table 1:** Baseline characteristics of 72 patients with locally advanced head and neck squamous cell carcinoma according to whether they achieved pathological complete remission (pCR)

Characteristic	pCR (*n* = 25)		Non-pCR (*n* = 47)		*p*-value
	n	%	n	%	
Gender					
Male	22	35.5	40	64.5	0.735
Female	3	30.0	7	70.0	
Median age (years)	55		55		
Primary site					
Oral cavity	4	30.8	9	69.2	0.869
Oropharynx	4	30.8	9	69.2	
Larynx/Hypopharynx	17	37.0	29	63.0	
T stage					
T1	1	50.0	1	50.0	0.218
T2	7	63.6	4	36.4	
T3	7	35.0	13	65.0	
T4a	9	26.5	25	73.5	
T4b	1	20.0	4	80.0	
N stage					
N0	2	25.0	6	75.0	0.266
N1	1	10.0	9	90.0	
N2	18	40.9	26	59.1	
N3	4	40.0	6	60.0	
Clinical group					
III	1	14.3	6	85.7	0.463
IVA	9	22.5	31	77.5	
IVB	5	33.3	10	66.7	
Concurrent chemotherapy					
Yes	10	27.0	27	73.0	0.158
No	15	42.9	20	57.1	
Radiotherapy technique					
2D	14	29.8	33	70.2	0.228
IMRT	11	44.0	14	56.0	

*2D* Two dimensional, *IMRT* Intensity-modulated radiotherapy

### Univariate analysis of prognostic impact of pCR for treatment outcomes

**Fig. 1:**
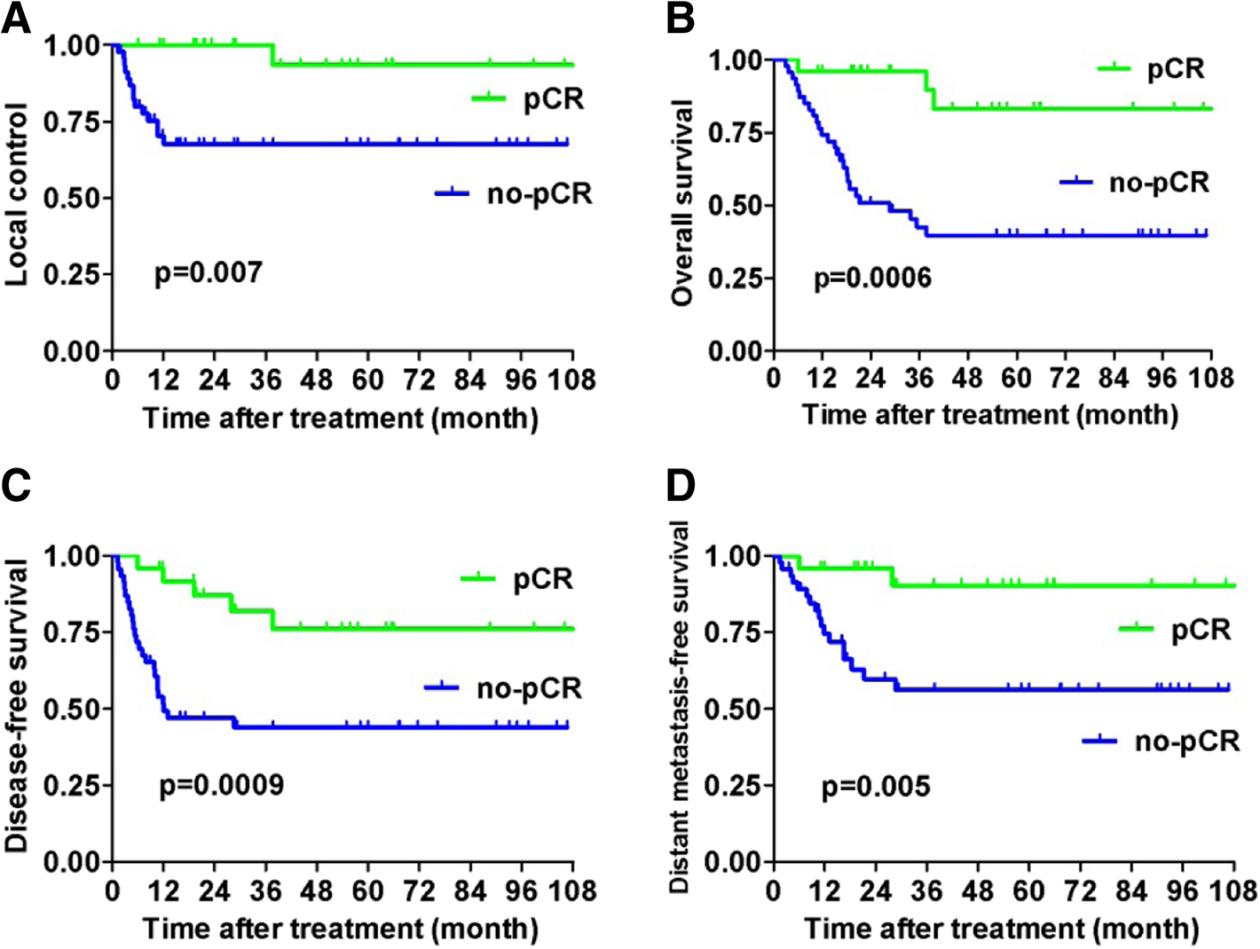
Differences in treatment outcomes between pCR and non-pCR patients among the whole cohort of patients: **a** local control, **b** overall survival, **c** progression-free survival, and **d** distant metastasis-free survival

Over a median follow-up of 24 months (range, 3–122 months), the 5-year estimated LC, OS, PFS, and DMFS for pCR patients and non-pCR patients were 93.2% vs. 67.7% (*p* = 0.007), 83.3% vs. 39.7% (*p* = 0.0006), 76.1% vs. 44.0% (*p* = 0.003), and 90.4% vs. 56.3% (*p* = 0.005), respectively (Fig. 1).

**Fig. 2:**
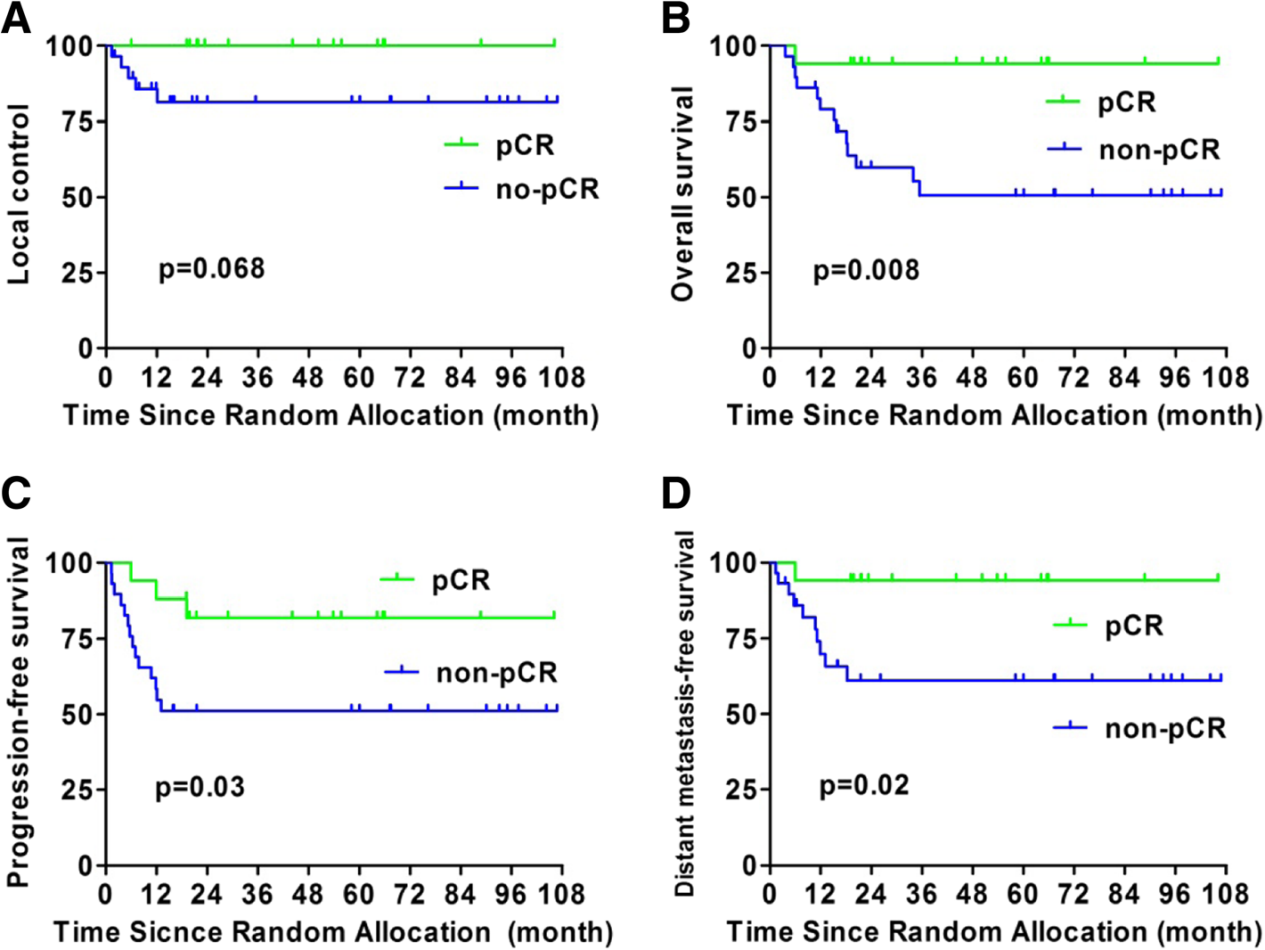
Differences in treatment outcomes between the pCR and non-pCR patients who had primary tumors of the larynx-hypopharynx: **a** local control, **b** overall survival, **c** progression-free survival, and **d** distant metastasis-free survival

There were 46 non-responders with larynx-hypopharynx primary tumors, 17 of whom achieved pCR of the primary lesion. The 5-year estimated LC, OS, PFS, and DMFS for these pCR and non-pCR patients were 100% vs. 81.4% (*p* = 0.068), 94.7% vs. 50.1% (*p* = 0.008), 81.9% vs. 51.2% (*p* = 0.03), and 94.1% vs. 61.1% (*p* = 0.02), respectively (Fig. 2).

### Multivariate analysis of prognostic impact of pCR for treatment outcomes

In multivariate analysis, pCR was an independent prognostic factors of LC(*p* = 0.002) (Table 2) and OSwith statistically significant differences (p<0.001) (Table 3). And pCR also played an significant role in DMFS and PFS (Additional file 1: Table S1). In addition, the degree of pathological differentiation, primary lesion in hypopharynx/larynx and N stage were also independent prognostic factors for OS. Other factors such as gender, age, T stage, technology of radiation and chemotherapy were not independent prognostic factors for OS.

**Table 2:** Multivariate analysis for the local control

		B	SE	p	HR (95% CI)
Gender	male vs female	−.181	.702	0.797	0.835 (0.211–3.304)
Age	≤55 vs >55	1.263	.730	0.084	3.535 (0.845–14.792)
Primary site	hypopharyngeal carcinoma/laryngeal carcinoma vs other primary sites	3.114	.847	0.000	22.503 (4.279–118.346)
Degree of pathological differentiation	Well/modarate vs poor differentiaon / undifferentiation	3.036	.914	0.001	20.828 (3.476–124.821)
T stage	T4 vs T1–3	−1.782	.757	0.019	0.168 (0.038–0.743)
N stage	N0 vs N1–2 vs N3	2.445	.809	0.003	11.527 (2.361–56.271)
Technology of radiation	2D vs IMRT	.334	.971	0.731	1.396 (0.208–9.359)
Chemotherapy	No vs Yes	−.182	.592	0.758	0.833 (0.261–2.662)
pCR	Yes vs No	4.004	1.290	0.002	54.839 (4.373–687.660)

**Table 3:** Multivariate analysis for the overall survival

		B	SE	p	HR (95% CI)
Gender	male vs female	−.816	.561	0.146	0.442 (0.147–1.327)
Age	≤55 vs >55	.359	.425	0.399	1.431 (0.622–3.294)
Primary site	hypopharyngeal carcinoma/laryngeal carcinoma vs other primary sites	1.024	.431	0.018	2.783 (1.196–6.476)
Degree of pathological differentiation	Well/modarate vs poor differentiaon / undifferentiation	1.833	.543	0.001	6.254 (2.157–18.129)
T stage	T4 vs T1–3	.406	.441	0.357	1.501 (0.633–3.564)
N stage	N0 vs N1–2 vs N3	1.511	.489	0.002	4.532 (1.737–11.824)
Technology of radiation	2D vs IMRT	−.256	.572	0.654	0.774 (0.252–2.376)
Chemotherapy	No vs Yes	−.596	.419	0.155	0.551 (0.242–1.252)
pCR	Yes vs No	2.460	.694	0.000	11.71 (3.003–45.663)

## Discussion

The current analysis revealed that clinical outcomes including local control, OS, PFS, and DMFS were statistically better in patients with locally advanced HNSCC who achieved pCR than in those who did not. These results indicate that pCR could be used as a potential prognostic factor for patients with locally advanced HNSCC after preoperative irradiation.

Non-response after preoperative treatment was defined by a reduction in the primary lesion of less than 80% at the end of 50 Gy irradiation with or without chemotherapy as evaluated by CT/MRI and endoscopy examination. We identified 25 out of 72 (34.7%) patients who achieved pCR among those who were non-responders to preoperative treatment. In the study by Kirita et al. [12], 48 patients with oral cavity cancer received cisplatin- or carboplatin-based preoperative concurrent chemoradiotherapy (RT 40 Gy), and the clinical CR and pCR rates were 60.4 and 50%, respectively. The lower pCR rate found in our study can primarily be attributed to the fact that only non-responding patients received surgical treatment in our study. In our phase 3 study, of the 222 patients enrolled, only 72 were non-responders based on imaging examination after 50 Gy irradiation with or without chemotherapy and underwent surgery. The other 150 patients who showed a good response received non-surgical therapy, and it is reasonable to expect that if they had been treated with surgery, the pCR rate would be higher in the 150 good responders than in the 72 non-responders.

Although planned preoperative concurrent chemoradiotherapy in head and neck cancer is not as popular as in esophageal cancer, rectal cancer, breast cancer, and non-small cell lung cancer, the relationship between pathological response and clinical outcome after preoperative radiotherapy has also been well studied. Friesland et al. [13] reported that among 167 patients with tonsillar carcinoma treated by radiotherapy with or without surgery, 28% of patients received surgery for the primary site and/or neck dissection after radiotherapy, and the 5-year OS for patients with pCR was 43%, whereas that for patients with non-pCR was 9% (*p* < 0.0001). The long-term prognostic value of achieving clinical CR, especially pCR in oral cancer was reported by Kirita et al [12], who found that the 10-year PFS of patients with pCR was better than that of patients with extensive residual tumor (87.5% vs. 40%). In addition, patients who showed good histopathology responses had superior survival (*p* = 0.012). The same results were found in patients with tongue carcinoma [14]; the PFS rates according to tumor regression rate were 33.3% for patients with less than 50% tumor regression, 66.7% for patients with 50–75% regression, 100% for patients with 75–100% regression, and 96.0% for patients with complete regression. The survival rate was statistically different between patients who achieved a regression rate of 75% or higher and those who did not (*p* < 0.0001). Other authors also reported significant differences in treatment outcomes between good and poor responders, with 5-year survival rates of 68–84% versus 24–32% [10, 15].

Recently, preoperative concurrent chemoradiotherapy has been used increasingly more frequently in esophagogastric cancer, non-small cell lung cancer, rectal cancer, and breast cancer, and it was found that a good pathologic response to preoperative chemoradiotherapy correlates with better long-term survival in these cancers [16–25]. Even in resectable non-small cell lung cancer, the major pathological response was suggested to be a surrogate endpoint for survival in future neoadjuvant trials [23]. Based on the results obtained in studies of esophagogastric cancer and rectal cancer, planned preoperative concurrent chemoradiotherapy may play an important role in the treatment of locally advanced head and neck cancer.

The relationship between clinical response and pathological response is still controversial. In our study, 25 (34.7%) clinical non-responders’ achieved pCR for the primary lesion. For gastric-esophagus conjunction carcinoma, Cheedella et al. [18] reported that the specificity of clinical CR for pCR is too low to be used for clinical decision-making regarding the delay or avoidance of surgery. The same finding was reported in rectal cancer treated with preoperative chemoradiotherapy [26]. Accurately predicting pCR after preoperative treatment and thereby avoiding surgery is difficult. This issue has been the focus of many efforts in recent years, and functional imaging has been suggested to resolve this problem. Hatakenaka et al. [27] found that pretreatment apparent diffusion coefficient (ADC) of the primary lesion correlated with local failure in 38 primary HNSCC patients treated with chemoradiotherapy or radiotherapy. Vandecaveye et al. [28] compared the change in ADC between before and 3 weeks after CRT (ΔADC) in 29 HNSCC patients and found that the negative predictive value of ΔADC in terms of tumor response for the primary lesion was 100%, and diffusion-weighted imaging (DWI) was better than anatomical imaging in predicting tumor response. Jacobs et al. [29] also found that the ΔADC during chemoradiotherapy and 4 weeks post-chemoradiotherapy were the best predictive parameters for pathological good response. Ceulemans et al. [30] investigated the role of fluorodeoxy glucose positron emission tomography PET/CT during radiotherapy (47 Gy) and 4 months after radiotherapy in 40 HNSCC patients and found that PET/CT at both times had a high specificity and positive predictive value for the evaluation of tumor response, suggesting that it might be used as an indicator for avoiding unnecessary salvage surgery in patients with CR, although PET/CT at 4 months after radiotherapy had the strongest predictive power. Hur et al. [31] reported a biomarker-based scoring system for predicting tumor response after preoperative chemoradiotherapy in rectal cancer. They found that the mRNA expression levels of four biomarkers (p53, p21, Ki67, and CD133) significantly correlated with tumor regression grade and pathologic complete response. Radiogenomics, which links different imaging features with diverse genomic events, is a new and exciting field within radiology, and imaging genomic linkages can help in monitoring treatment response. This method now is widely investigated in many types of tumors such as brain tumor, rectal cancer, and head and neck cancer [32, 33].

In summary, for locally advanced HNSCC, a multidisciplinary treatment modality was the mainstay treatment choice. Achievement of pCR after preoperative treatment is associated with good treatment outcomes. At present, induction chemotherapy followed by concurrent chemoradiotherapy or concurrent chemoradiotherapy, with surgery as salvage therapy, is used with increasing frequency in the treatment of locally advanced HNSCC. Determining how to assess the tumor response accurately after preoperative treatment is very important, and for patients who achieved pCR, surgery may be avoidable, which improves organ function preservation. Moreover, it is reasonable to expect that the tumor response after preoperative treatment will help to predict outcome even more accurately when combined with clinical characteristics based on functional imaging, biomarkers, and genomics.

## Supplementary information

**Additional file 1: Table S1.** Multivariate analysis for DMFS and PFS.

## Data Availability

The datasets used and/or analyzed during the current study are available from the corresponding author on reasonable request.

## Abbreviation

2D: Two dimensional
ADC: Apparent diffusion coefficient
CR: Complete remission
CT: Computed tomography
DMFS: Distant metastasis-free survival
DWI: Diffusion-weighted imaging
HNSCC: Head and neck squamous cell carcinoma
IMRT: Intensity-modulated radiotherapy
LC: Local control
MRI: Magnetic resonance imaging
mRNA: messenger RNA
NCCN: National Comprehensive Cancer Network
OS: Overall survival
pCR: pathological complete remission
PET: Positron emission tomography
PFS: Progression-free survival
