# Supplementary material for: Prognostic impact of pathological complete remission after preoperative irradiation in patients with locally advanced head and neck squamous cell carcinoma: re-analysis of a phase 3 clinical study
Source: Radiat Oncol. 2019 Dec 12;14:225. doi: 10.1186/s13014-019-1428-4 (PMC6909460; doi:10.1186/s13014-019-1428-4)
Supplement: Supplementary file 1 — Additional file 1: Table S1. Multivariate analysis for DMFS and PFS. [file 13014_2019_1428_MOESM1_ESM.docx]

Additional file 1: Table S1. Multivariate analysis for DMFS and PFS

| Variates | | PFS | | DMFS | |
| --- | --- | --- | --- | --- | --- |
|  |  | p | HR（95%CI） | p | HR（95%CI） |
| Gender | male vs female | 0.696 | 0.813  （0.287-2.299） | 0.633 | 0.705  （0.167-2.968） |
| Age | ≤55 vs ＞55 | 0.450 | 1.360  （0.613-3.017） | 0.660 | 0.794  （0.284-2.219） |
| Primary site | hypopharyngeal carcinoma/ laryngeal carcinoma vs other primary sites | 0.215 | 1.689  （0.737-3.868） | 0.866 | 1.096  （0.376-3.196） |
| Degree of pathological differentiation | Well/modarate vs poor differentiation / un-differentiation | 0.034 | 2.862  （1.084-7.560） | 0.498 | 1.559  （0.431-5.641） |
| T stage | T4 vs T1-3 | 0.723 | 1.166  （0.499-2.727） | 0.926 | 1.050  （0.376-2.933） |
| N stage | N0 vs N1-2 vs N3 | 0.197 | 1.731  （0.752-3.986） | 0.629 | 1.303  （0.445-3.812） |
| Technology of radiation | 2D vs IMRT | 0.189 | 0.514  （0.190-1.388） | 0.585 | 0.703  （0.198-2.496） |
| Chemotherapy | No vs Yes | 0.195 | 0.594  （0.270-1.306） | 0.103 | 0.435  （0.160-1.185） |
| pCR | Yes vs No | 0.002 | 5.230  （1.79-15.25） | 0.010 | 7.637  （1.614-36.13） |

Annotation: In multivariate analysis for PFS, the degree of pathological differentiation and pCR were the independent prognostic factors. And for DMFS, only pCR had significant effect on DMFS.
